# Supplementary material for: A study of UK household wealth through empirical analysis and a non-linear Kesten process
Source: PLoS One. 2022 Aug 24;17(8):e0272864. doi: 10.1371/journal.pone.0272864 (PMC9401122; doi:10.1371/journal.pone.0272864)
Supplement: S1 Appendix — (PDF) [file pone.0272864.s001.pdf]

Supporting information

SI.1 Theory

SI.1.1 Mean and variance of returns

With R_n defined as in (2) and α_n i.i.d. from some distribution with $\mu = \mathbb{E}[\alpha_{n+1}]$ and $\sigma^2 = \mathrm{var}(\alpha_{n+1})$ as in Section 2 we have

$$\mathbb{E}[R_{n+1}|W_n] = \mathbb{E}[\alpha_{n+1}W_n^{\gamma-1}|W_n] = \mathbb{E}[\alpha_{n+1}]W_n^{\gamma-1} = \mu W_n^{\gamma-1}, \tag{13}$$

$$\mathrm{var}[R_{n+1}|W_n] = \mathrm{var}[\alpha_{n+1}W_n^{\gamma-1}|W_n] = \mathrm{var}[\alpha_{n+1}]W_n^{2(\gamma-1)} = \frac{\sigma^2}{\mu^2}\mathbb{E}[R_{n+1}|W_n]^2. \tag{14}$$

Thus the interval of one standard deviation around the mean of R_{n+1} used in Figure 2 is

$$\Big(\Big(1-\frac{\sigma}{\mu}\Big)\mathbb{E}[R_{n+1}|W_n], \Big(1+\frac{\sigma}{\mu}\Big)\mathbb{E}[R_{n+1}|W_n]\Big) = \Big((\mu-\sigma)W_n^{\gamma-1}, (\mu+\sigma)W_n^{\gamma-1}\Big). \tag{15}$$

SI.1.2 Linear Kesten process

Consider a general linear Kesten process

$$W_{n+1} = A_{n+1}W_n + B_{n+1}\ , \quad n = 0, 1, 2, \dots\ , \tag{16}$$

so that we can write

$$W_n = \prod_{k=1}^{n} A_k \left(W_0 + \sum_{k=1}^{n} B_k \prod_{i=1}^{k} A_i^{-1} \right). \tag{17}$$

Here $(A_n)_n$ and $(B_n)_n$ are sequences of i.i.d. random variables with

$$\mu := \mathbb{E}\big[\log|A_k|\big] \in \mathbb{R} \quad \text{and} \quad \nu^2 := \mathrm{Var}\big[|\log A_k|\big] \in (0,\infty). \tag{18}$$

$\boldsymbol{\mu < 0}$. For the stationary case, Kesten proved the following result, exact details can be found in Theorem 5 in [1]: if $\mu < 0$, there exists $\beta > 0$ such that $\mathbb{E}[|A_n|^\beta] = 1$ and provided several other mild regularity conditions on the distributions of A_n and B_n are satisfied,

$$W_n \to W_\infty := \sum_{k=1}^{\infty} B_k \prod_{i=1}^{k-1} A_i \quad \text{in distribution as } n \to \infty, \tag{19}$$

for all initial conditions W_0. The stationary distribution of W_∞ exhibits a power law in one or both tails with parameter β, i.e. the following limits

$$\lim_{w\to\infty} w^\beta \mathbb{P}(W_\infty > w) \quad \text{and} \quad \lim_{w\to\infty} w^\beta \mathbb{P}(W_\infty < -w), \tag{20}$$

exist and are finite, with at least one of them strictly greater than zero.

$\boldsymbol{\mu > 0}$. Following recent results in [2], this non-stationary case can be analysed as follows. Taking absolute values and logarithms in (17) we get

$$\frac{\log|W_n| - \mu n}{\sqrt{n}\nu} = \frac{\sum_{k=1}^{n}\log|A_k| - \mu n}{\sqrt{n}\nu} + \frac{\log\left(\left|W_0 + \sum_{k=1}^{n} B_k \prod_{i=1}^{k} A_i^{-1}\right|\right)}{\sqrt{n}\nu}. \tag{21}$$

By the CLT for i.i.d. random variables A_k we have

$$\frac{\sum_{k=1}^{n}\log|A_k| - \mu n}{\sqrt{n}\nu} \to \mathcal{N}(0,1) \quad \text{in distribution as } n \to \infty.$$

Since $\mathbb{E}[\log|A_i|^{-1}] = -\mu < 0$, we have $\left|W_0 + \sum_{k=1}^{n} B_k \prod_{i=1}^{k} A_i^{-1}\right| \xrightarrow{n\to\infty} \widetilde{W}_\infty < \infty$, corresponding to the limit in the stationary case. This implies $\frac{1}{\sqrt{n}\nu} \log\left(\left|W_0 + \sum_{k=1}^{n} B_k \prod_{i=1}^{k} A_i^{-1}\right|\right) \to 0$, and with (21)

$$\frac{\log|W_n| - \mu n}{\sqrt{n}\nu} \to Z \sim \mathcal{N}(0,1) \quad \text{in distribution as } n \to \infty. \tag{22}$$

This implies that

$$\frac{|W_n|^{1/(\sqrt{n}\nu)}}{e^{\mu\sqrt{n}/\nu}} \xrightarrow{d} e^Z \sim \text{Lognorm}(0,1) \quad \text{in distribution as } n \to \infty.$$

Using the natural scale invariance of (17) we get a linear dependence on the initial condition, and to leading exponential order (21) as $n \to \infty$

$$|W_n| \simeq |W_0| \exp(\mu n + \sqrt{n}\nu Z).$$

We note that [2] also includes the case with $\mu = 0$ which we do not discuss here.

SI.1.3 Non-linear Kesten process

We analyse the non-linear process with $\gamma > 1$ given in (3) as

$$W_{n+1} = W_n + \alpha_{n+1} W_n^\gamma + S_{n+1}.$$

For simplicity we assume $\alpha_n > 0$, which implies that W_n is increasing and strictly positive for all $n \geq 0$. Negative values of α_n will lead to bankruptcy events as $n \to \infty$, for which we apply replacement mechanisms (**R.1**)-(**R.3**) as explained in Section 4. Taking logarithms leads to

$$\begin{aligned} \log W_{n+1} &= \log\left(W_n + \alpha_{n+1} W_n^\gamma + S_{n+1}\right) \\ &= \gamma \log W_n + \log\left(\alpha_{n+1} + 1/W_n^{\gamma-1} + S_{n+1}/W_n^\gamma\right) \\ \text{so that} \quad X_{n+1} &= \gamma X_n + B_{n+1}, \end{aligned} \tag{23}$$

where $X_n := \log W_n$ and $B_{n+1} := \log(\alpha_{n+1} + 1/W_n^{\gamma-1} + S_{n+1}/W_n^\gamma)$. Now using (17) we get

$$X_n = \gamma^n \Big(X_0 + \sum_{k=1}^{n} B_k \gamma^{-k}\Big) \quad \text{so that} \quad \frac{X_n}{\gamma^n} \xrightarrow{d} X_0 + D \quad \text{as } n \to \infty, \tag{24}$$

where $D := \sum_{k=1}^{\infty} B_k \gamma^{-k}$. Since $W_n > 0$ is increasing with n and α_n are i.i.d., B_k are bounded random variables, so $D \in (0,\infty)$ is a well defined random variable since $\gamma > 1$. Thus, as $n \to \infty$, this implies to leading exponential order

$$\frac{X_n}{\gamma^n} \simeq X_0 + D \quad \text{so that} \quad W_n \asymp \left(W_0 e^D\right)^{\gamma^n}. \tag{25}$$

SI.2 Empirical analysis

SI.2.1 Data sources

Here we list the data sources used in the paper:

1. Biannual wealth and asset survey (WAS) data 2008-2016 from the Office for National Statistics (ONS) [3]

2. Forbes rich lists [4]

3. Times rich list data - extracted from Times online newspaper 2019, 2020 and 2021. See [5] for this data and for the current list see [6].

4. ONS household income, salary and expenditure data [7, 8]

Full details on the data can be found at the author's repository [5].

SI.2.2 Notation for tails and power laws

A random variable $X \geq 0$ exhibits a power-law tail with exponent $\beta > 0$ if

$$\mathbb{P}(X > x) \simeq \frac{C}{x^\beta} \quad \text{for some } C > 0 \text{ to leading order as } x \to \infty.$$

The Pareto distribution with scale parameter $x_m > 0$ is a standard example for power-law tails, where

$$X \sim \text{Pareto}(x_m, \beta) \quad \text{if} \quad \mathbb{P}(X > x) = \left(\frac{x_m}{x}\right)^\beta \quad \text{for } x \geq x_m.$$

We can estimate the tail of a random variable X by the empirical tail $\mathbb{P}_N(X > x)$ of a random sample $\{x_1, x_2, \ldots, x_N\}$ of X defined as

$$\mathbb{P}_N(X > x) := \frac{1}{N} \sum_{i=1}^{N} \mathbf{1}_{x_i > x}$$

where $\mathbf{1}$ is the indicator function. It is a standard result that $\mathbb{P}_N(X > x) \to \mathbb{P}(X > x)$ almost surely as $N \to \infty$.

To fit the power-law tail parameters C and β we use linear regression in a window of interest on the double logarithmic scale $\log \mathbb{P}_N(X > x)$ vs. $\log x$. This simple technique is known to introduce a bias in the fit [9, 10], but for our purposes in this paper we find it is a sufficient approximation.

SI.2.3 Tail of UK wealth

Here we outline how we extract the empirical tail from wealth survey and rich list data from [3, 4]. For extensive discussion on the WAS see [11]. We have wealth survey data in the form $(\tilde{h}_i, \tilde{w}_i)$ for $i = 1, 2, \ldots n$ where $\tilde{h}_i \in [0, 1]$ is the cumulative proportion of households and $\tilde{w}_i \in [0, 1]$ is their corresponding cumulative proportion of wealth. Let the data be ordered with increasing wealth per household, then the the Gini coefficient can be calculated from the Lorenz curve [3] defined by the points $(\tilde{h}_i, \tilde{w}_i)$. Let H and W be the total number of households and the total amount of wealth of all households respectively.

Define $\hat{w}_i := (\tilde{w}_{i+1} - \tilde{w}_i)W$ and $\hat{h}_i := (\tilde{h}_{i+1} - \tilde{h}_i)H$ for $i = 1, 2, \ldots, n-1$. Then $\hat{w}_i$ is the total amount of wealth owned by an increasingly rich number $\hat{h}_i$ of households, so that $w_i := \frac{\hat{w}_i}{\hat{h}_i}$ is the corresponding average amount. Since the original data was ordered we have also $w_i \leq w_{i+1}$ for all i. Therefore the points $(w_i, \tilde{h}_{i+1})$ characterise an approximation to the empirical CDF and the points $(w_i, 1 - \tilde{h}_{i+1})$ give the corresponding approximation to the empirical tail of the wealth distribution. We plot the empirical tail of positive wealth in Figure 1 which are points below £10^8 for the years 2008, 2010, 2012, 2014 and 2016.

We have separate data in the form of rich lists for individual household wealth w_i for $i = 1, 2, \ldots, R$, where R are the number of households in the rich list. We assume the

rich list contains the R wealthiest households in the population, so for ordered w_i the empirical tail of their wealth distribution is given by the points $(w_i, (R-i)/H)$. The empirical tail of the rich lists are the points above £10^8 in Figure 1, and are matched in colour for corresponding years to the survey data.

SI.2.4 Estimating the ROR

To approximate returns of individual household wealth using the WAS data [3] we use the returns on percentile wealth. For each time period n from the empirical tail of the survey we extract the percentile $w_{i,n}$ such that $\mathbb{P}_N(W_n > w_{i,n}) = p_i$ where $p_i = 1 - i/100$ for $i \in \{1, 2, \ldots, 100\}$. Note we only extract positive percentiles i such that $w_{i,n} > 0$, which excludes the poorest households. Then we substitute $W_n = w_{i,n}$ in (7) to calculate RORs of percentiles over each of the five biannual time periods 2008-2016 of the data [3]. For percentile i we have the ROR as

$$r_{i,n+2} = \frac{w_{i,n+2} - w_{i,n} - 2s_{i,n+2}}{2w_{i,n}}, \tag{26}$$

where $s_{i,n}$ are the yearly savings in percentile i and time period n (see Section 3.3 for details).

Note that these percentile RORs for the ONS survey data [3] plotted in Figure 2 only approximate RORs for individual households. Our procedure does not account for households changing percentiles over a time period, leading to reduced fluctuations of the resulting returns data. To compensate for this and also possible effects of the financial crisis from 2008 onwards, we combine all time periods in a single data set to infer system parameters. For billionaires we have individual wealth data across time. We ignore residual savings to compute returns according to (7), and plot these values for 2016 in Figure 2.

In order to understand the dependence of ROR on wealth in particular for the UK, it is instructive to consider the different composition of wealth for poorer and richer households. Survey data [3] differentiate four components of wealth: property, physical, financial and pension, and their typical distribution is summarised in Figure S1 Fig, exemplary for 2016 data. Financial and property wealth of the poorest decile have a negative sign (i.e. constitute debt), and the total average wealth in that decile is approximately 0 and not shown in Figure S1 Fig.

The paper 'The rate of return on everything, 1870–2015' [12] provides a comprehensive analysis of average returns across four different types: bills, bonds, equity and housing over 1870-2015. In particular for the period 1980-2015 the average real rate of returns on equity and housing for the UK are 9.11% and 6.81%, respectively (Table 7, p 37 [12]). Therefore, the increasing proportion of property and financial wealth for wealthier households can account for RORs increasing with wealth. This is also confirmed in Figure S1 Fig (bottom), where we see that ROR (technically ROR with zero residual savings as it is unclear how to divide savings across components) for physical and pension wealth are largely independent of wealth, while property and financial ROR increase with wealth.

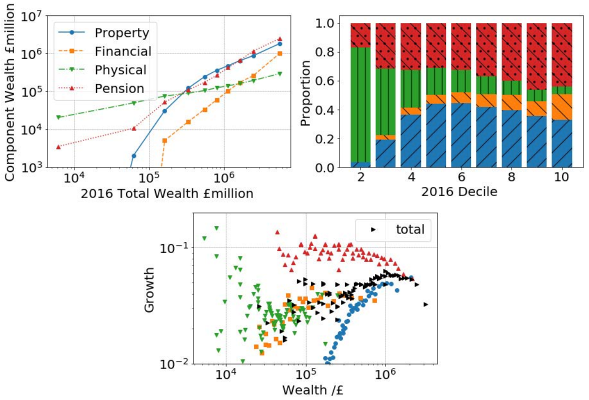

S1 Fig. Absolute wealth by components as a function of total wealth (top left) and wealth proportions by component of positive wealth deciles (top right), both from ONS data [3] from 2016. Bottom: ROR with zero savings (wealth growth) and same colour code averaged over time periods from 2008 to 2016, computed as described in (26) from percentile data [3].

SI.2.5 Inequality measures

There are several measures of inequality each with their various merits [13, 14]. In this paper we use the standard Gini coefficient $g \in [0, 1]$ and top 1% wealth share $s_{0.01} \in (0, 1]$. The Gini coefficient can be thought of as a measure of the difference between any two randomly selected agents' wealth. The top 1% wealth share is defined as the proportion of wealth held by the richest 1% of the population.

For a non-decreasing ordered sample of N agents' wealth $w_1 \leq w_2 \leq \cdots \leq w_N$ with total wealth $W = \sum_{i=1}^{N} w_i$ we define

$$s_{0.01} := \sum_{i>0.99N}^{N} w_i \Big/ W \quad \text{and} \quad g := \frac{2}{N} \sum_{i=1}^{N} i w_i \Big/ W - \frac{N+1}{N}. \tag{27}$$

We note the two extreme cases:

1. Perfect equality: $w_1 = w_2 = \cdots = w_N \Rightarrow g = 0$ and $s_{0.01} = 0.01$;
2. Perfect inequality: $w_i = 0$ for $i = 1, 2, \ldots, N-1$ and $w_N > 0 \Rightarrow g = 1$ and $s_{0.01} = 1$.

The UK top one percent wealth share (see Figure S2 Fig) has decreased significantly from 1895 until around 1985, and is increasing slightly since then.

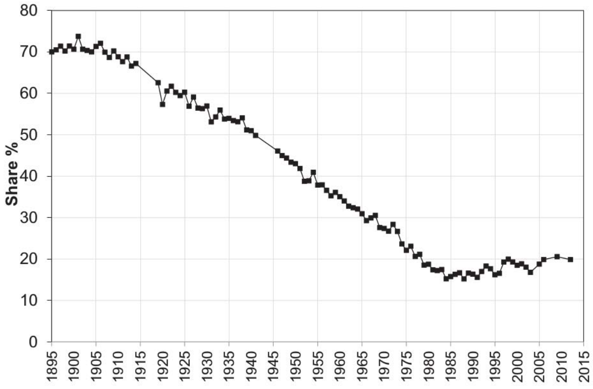

S2 Fig. Top 1% UK wealth share from 1895-2013 taken from [15]

SI.2.6 Non-central t distribution

We fit the coefficients α_n (10) with a shifted and scaled non-central t-distribution (nct) as seen in Figure 4, which has been used for fitting stock returns that are both skewed and heavy tailed [16]. The standard non-central t distribution is defined by a random variable

$$U = \frac{Z + c}{\sqrt{V/k}}\ , \quad \text{where } Z \sim \mathcal{N}(0,1) \text{ is a standard Gaussian,}$$

$c \in \mathbb{R}$ is the centrality parameter, and $V \sim \chi^2(k)$ with $k > 0$ the degrees of freedom. The shifted and scaled non-central t-distribution we use is then given by the random variable

$$sU + l \sim \text{nct}(k, c, l, s),$$

with the shift parameter $l \in \mathbb{R}$ and scale parameter $s > 0$.

SI.3 Supplementary simulation results

SI.3.1 Generic initial conditions

Recall the replacement mechanisms (**R.1**)-(**R.3**) in case of bankruptcy events:

R.1 replace with a proportion of the agent's previous positive wealth value $pW_{n-1}(i) > 0$ such that p is uniformly chosen from $(0, 1]$

R.2 replace with the agent's previous positive wealth value $W_{n-1}(i) > 0$

R.3 replace with wealth $W_n(j) > 0$ of another uniformly chosen agent j

We can see from Figures 6, 9 (top left and right), and S3 Fig and S4 Fig below that the empirical tails and inequality measures of the simulations (3) evolve similarly in time for the three replacement mechanisms (**R.1**)-(**R.3**) until the system enters the crossover region for wealth values around 10^{14} and time about $n = 200$. Then bankruptcy events become more frequent and relevant for the richest households, leading to significant differences with mechanism **R.3** naturally leading to slowest growth.

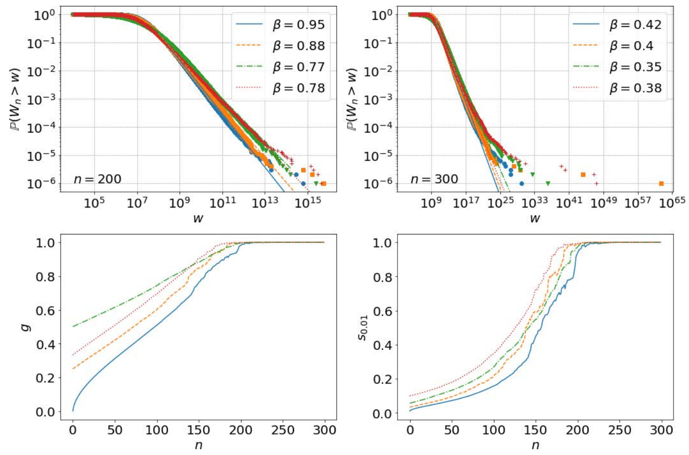

S3 Fig. Simulation (3) with $N = 10^6$ agents, residual savings $S_n = 0$, $\alpha_n \sim \text{nct}(k, c, l, s)$ with fitted parameters in (12) and $\gamma = 1.075$ for the four initial conditions with colours and symbols as in **I.1-I.4** and replacement mechanism **R.2**. Top left and right show empirical tails at times $n = 200, 300$ and power-law tail fits with exponents β. Bottom left and right show respective Gini, g, and top 1% wealth shares, $s_{0.01}$ up to $n = 300$ with initial conditions **I.1** (full line), **I.2** (dashed), **I.3** (dash-dotted) and **I.4** (dotted).

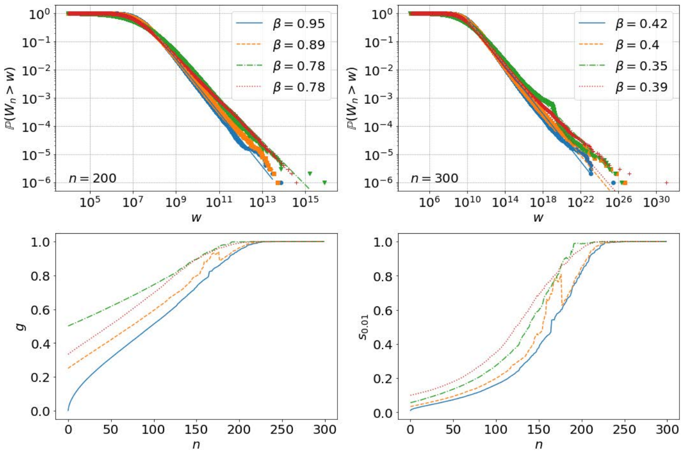

S4 Fig. Simulation (3) with $N = 10^6$ agents, residual savings $S_n = 0$, $\alpha_n \sim \text{nct}(k, c, l, s)$ with fitted parameters in (12) and $\gamma = 1.075$ for the four initial conditions with colours and symbols as in **I.1**-**I.4** and replacement mechanism **R.3**. Top left and right show empirical tails at times $n = 200, 300$ and power-law tail fits with exponents β. Bottom left and right show respective Gini, g, and top 1% wealth shares, $s_{0.01}$ up to $n = 300$ with initial conditions **I.1** (full line), **I.2** (dashed), **I.3** (dash-dotted) and **I.4** (dotted).

SI.3.2 Realistic initial conditions

We can see from Figures 10, S5 Fig and S6 Fig that the three replacement mechanisms (**R.1**)-(**R.3**) give very similar results on wealth distribution and inequality over time n, for the simulations described in the caption of Figure S5 Fig. This is due to the much shorter time horizon compared to our numerical studies of generic initial conditions, and confirms that the choice of replacement mechanism is not crucial over limited time periods.

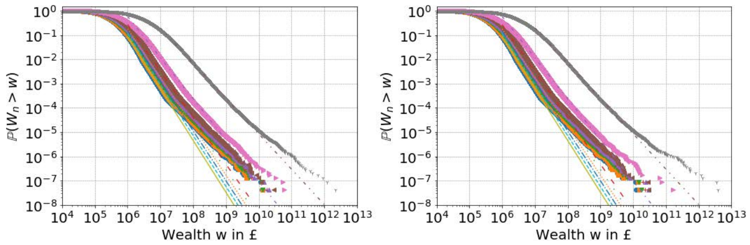

S5 Fig. Empirical tails for simulation (3) with $N \approx 23 \cdot 10^6$ agents, replacement mechanisms **R.2** (left) and **R.3** (right), fixed residual savings $S_n = S(W_0)$ (11), $\alpha_n \sim \text{nct}(k, c, l, s)$ with fitted parameters in (12) and $\gamma = 1.075$ for 2008 initial conditions at times $n = 0, 2, 4, 6, 8, 10, 20$ and 50. Power law fits with exponents β decreasing from $\beta = 2.13$ at $n = 0$ to $\beta = 1.45$ at $n = 50$.

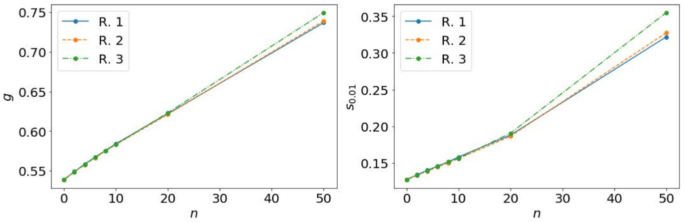

S6 Fig. Gini, g, (left), top 1% wealth shares, $s_{0.01}$, (right) for simulation (3) with $N \approx 23 \cdot 10^6$ agents, fixed residual savings $S_n = S(W_0)$, $\alpha_n \sim \text{nct}(k, c, l, s)$ with fitted parameters (12) and $\gamma = 1.075$ for 2008 initial conditions and replacement mechanisms **R.1-R.3**.

References

1. Kesten H. Random difference equations and renewal theory for products of random matrices. Acta Mathematica. 1973;131(1):207–248.
2. Hitczenko P., Wesołowski J., et al. Renorming divergent perpetuities. Bernoulli. 2011;17(3):880–894.
3. ONS. Wealth and Assets Survey (WAS) Data;. See previous versions for granulated data. Accessed: 2019. `https://www.ons.gov.uk/peoplepopulationandcommunity/personalandhouseholdfinances/incomeandwealth/datasets/totalwealthwealthingreatbritain/july2006tojune2016`.
4. Bresler A. Forbes rich list data;. Accessed 2020. `https://github.com/abresler/forbesListR`.
5. Forbes S. Author's repository of saved data. `https://github.com/saf92/PLOS-ONE-Kesten`.
6. Times. Time dependent Times UK rich list data;. Accessed 2022. `https://www.thetimes.co.uk/sunday-times-rich-list#TableFullRichList`.
7. ONS. Income Data;. Accessed: 2019. `https://www.ons.gov.uk/peoplepopulationandcommunity/personalandhouseholdfinances/incomeandwealth/datasets/householddisposableincomeandinequality`.
8. ONS. Expenditure Data;. Accessed: 2019. `https://www.ons.gov.uk/peoplepopulationandcommunity/personalandhouseholdfinances/expenditure/datasets/detailedhouseholdexpenditurebydisposableincomedecilegroupuktable31`.
9. Newman MC. Regression Analysis of Log-transformed Data: Statistical Bias and it's Correction. Environmental Toxicology and Chemistry, Vol 12, pp 1129-1133. 1993;.

10. Clauset A, Shalizi CR, Newman ME. Power-law distributions in empirical data. SIAM review. 2009;51(4):661–703.

11. ONS. Main Results from the Wealth and Assets Survey 2006/08. 2009;.

12. Jordà Ò, Knoll K, Kuvshinov D, Schularick M, Taylor AM. The rate of return on everything, 1870–2015. The Quarterly Journal of Economics. 2019;134(3):1225–1298.

13. Piketty T. Capital in the 21st Century. Harvard University Press; 2013.

14. Champernowne DG. A Comparison of Measures of Inequality of Income Distribution. The Economic Journal , Dec, 1974, Vol 84, No 336, pp 787-816. 1974;.

15. Alvaredo F, Atkinson AB, Morelli S. Top wealth shares in the UK over more than a century. Journal of Public Economics. 2018;162:26–47.

16. Tsionas EG. Bayesian inference in the noncentral Student-t model. Journal of Computational and Graphical Statistics. 2002;11(1):208–221.
