# Supplementary material for: A study of UK household wealth through empirical analysis and a non-linear Kesten process
Source: PLoS One. 2022 Aug 24;17(8):e0272864. doi: 10.1371/journal.pone.0272864 (PMC9401122; doi:10.1371/journal.pone.0272864)
Supplement: Attachment — Submitted filename: Response_to_referees.pdf [file pone.0272864.s003.pdf]

Response to Referees:

Referee #1:

We thank the referee for the useful comments, which we have adopted as follows:

1) In the introduction section, at least a paragraph about agent-based models should be added.

We have added a short explanation of the agent-based approach in an appropriate place in the introduction on pages 1 and 2.

2) I would suggest the authors add a new section “Data” to describe the empirical data used in the manuscript. Moreover, in this section, a Table reporting the main statistical properties of the data could be useful.

We renamed Section 3 “Empirics” to “Data analysis”, since we think that it contains all the relevant statistical properties of the data that are relevant for the paper. The most important properties are summarized in Figures 1 and 2, and we were not sure how to add more relevant information in a table. After careful consideration we decided to leave those figures in place at the beginning of the paper, in order to motivate our choice of the model. Further technical details on data and the methodology to analyse them is given in the supplementary material in Section SI.2 Empirical analysis.

3) The authors could add a new section “conclusions”, where the main conclusions and the practical implication of the study are presented.

We think that the content for the proposed section is present in 5 Discussion, which we renamed to 5 Conclusions.

4) Please improve the quality of the figures, use not only different colors to plot the curves but also different line style or marker so that also in black and white the figures are readable.

The quality of the figures has been improved as suggested. All Figures have been updated so they can now be differentiated in black and white using different line styles and markers.

Referee #2:

We thank the referee for carefully reading the manuscript and the useful comments, which we have adopted as follows:

3.1 Line 8 wealth/income shares. This expression using "/" is not clear sometimes. I suggest a verbal expression here.

Implemented as: … as well as wealth or income shares,

3.2 Line 99 "a simple model that can also be analysed analytically. " The term "be analysed analytically" seems pleonasm. Maybe, it can be replaced by be analysed using logical reasoning.

Changed to: … analysed mathematically. (which was the intended meaning)

3.3 Line 180 From (3) we rearrange to find the ROR. The resulting ROR is more relevant to the rearrangement from Equation (1).

Yes, we changed this as suggested.

3.4 L 89 Equation (1), L182 savings can typically be ignored, L213 We recall that in our model (1) savings S_n represent all contributions to wealth growth that are independent of the current wealth of an agent. Personally, there is a confusion about the meaning of the saving. From the expression in L213, we know that the saving is referred of as independent saving, a random valuable that doesn't affect the growth of wealth and its mean value can be captured using a logistic function. However, in the convectional macroeconomic context, saving is equal to investment, which is obviously different from the meaning of saving in this manuscript. Maybe, a "residual saving" or "independent saving", or the other word is a more accurate expression. Then, the residual saving makes sense in L182 that can be ignored comparing to the quantity of wealth. Consequently, please make it clear when giving Equation (1).

We have adopted this useful suggestion and now use the term "residual saving" throughout the manuscript.

3.5 Figure 6 Parameter β appeared once before Figure 6 as the parameter for Pareto distribution in I.4. What does β mean in Figure 6? Please avoid collision of notations, if β here suggests a power-law exponent.

We have removed β from Condition I.4 and only use it for exponents of power law fits in Figure 6 and later Figures.
