# Supplementary material for: A study of UK household wealth through empirical analysis and a non-linear Kesten process
Source: PLoS One. 2022 Aug 24;17(8):e0272864. doi: 10.1371/journal.pone.0272864 (PMC9401122; doi:10.1371/journal.pone.0272864)
Supplement: Attachment — Submitted filename: Reviewer Report_Zheng NAN.docx [file pone.0272864.s002.docx]

Peer review report on “A Study of UK Household Wealth through Empirical Analysis and a Non-linear Kesten Process”

1 Recommendation

Accept

2 Overview and general recommendation

This manuscript proposes a hypothetical iterative model to represent a generic dynamics of household wealth.

A tradeoff between wealth growth and wealth change has been made due to the assumption that “wealth exchange between households does not play an important role”, so the model keeps its simplicity while capturing the main features of wealth evolution.

The underlying hypothesis is that the rate of rate on the existing wealth follows a power-law form with a proportional random factor, $\alpha_{n}$, and an exponent, $\gamma$.

The significance of this study is that a positive value of $\gamma$ leads to a non-linear Kesten process, which is less studied in literature. Hence, the nonlinearity of the Kesten process provide a better explanation of the two-tail structure observed in the positive UK household wealth data.

Meanwhile, a comparison between a nonlinear Kesten process and a linear case ($\gamma=1$) has been employed, showing that the linear model causes the log-normal tail, but the non-linear models cause the power tails that are often observed in the dataset.

Moreover, the proposed framework focus on positive household wealth, however, it also nests the scenarios of a negative wealth increment and a bankruptcy, which are worthwhile further investigating in the future.

In my opinion, this manuscript is overall well written. The methodology is coherent with their assumption that “the rate of return on wealth is increasing with wealth” in the extremely wealthy scenario, though Keynesian liquidity preference theory suggests the negative relationship between the rate of return and the amount of investment. We can also observe that Keynesian theory is partially relevant in Figure 2 as the downward slopes present. I think it is an interesting topic for readers to discuss.

3 Minor comments

3.1

Line 8 wealth/income shares.

This expression using “/” is not clear sometimes. I suggest a verbal expression here.

3.2

Line 99 “a simple model that can also be analysed analytically. “

The term “be analysed analytically” seems pleonasm. Maybe, it can be replaced by be analysed using logical reasoning.

3.3

Line 180 From (3) we rearrange to find the ROR.

The resulting ROR is more relevant to the rearrangement from Equation (1).

3.4

L 89 Equation (1)

L182 savings can typically be ignored.

L213 We recall that in our model (1) savings $S_{n}$ represent all contributions to wealth growth that are independent of the current wealth of an agent.

Personally, there is a confusion about the meaning of the saving. From the expression in L213, we know that the saving is referred of as independent saving, a random valuable that doesn’t affect the growth of wealth and its mean value can be captured using a logistic function.

However, in the convectional macroeconomic context, saving is equal to investment, which is obviously different from the meaning of saving in this manuscript.

Maybe, a “residual saving” or “independent saving”, or the other word is a more accurate expression. Then, the residual saving makes sense in L182 that can be ignored comparing to the quantity of wealth. Consequently, please make it clear when giving Equation (1).

3.5

Figure 6 Parameter $\beta$ appeared once before Figure 6 as the parameter for Pareto distribution in I.4. What does $\beta$ mean in Figure 6? Please avoid collision of notations, if $\beta$ here suggests a power-law exponent.
